# Discovery of Potential Chemical Probe as Inhibitors of CXCL12 Using Ligand-Based Virtual Screening and Molecular Dynamic Simulation

**DOI:** 10.3390/molecules25204829

**Published:** 2020-10-20

**Authors:** Sajjad Haider, Assem Barakat, Zaheer Ul-Haq

**Affiliations:** 1 Dr. Panjwani Center for Molecular Medicine and Drug Research, International Center for Chemical Sciences, University of Karachi, Karachi 75270, Pakistan; sajjadrealist@gmail.com; 2 Department of Chemistry, College of Science, King Saud University, P. O. Box 2455, Riyadh 11451, Saudi Arabia; 3 Department of Chemistry, Faculty of Science, Alexandria University, P.O. Box 426, Ibrahimia, Alexandria 21321, Egypt

**Keywords:** chemokines, metastasis, cancer, pharmacophore based virtual screening, CXCR4, molecular dynamic simulation

## Abstract

CXCL12 are small pro-inflammatory chemo-attractant cytokines that bind to a specific receptor CXCR4 with a role in angiogenesis, tumor progression, metastasis, and cell survival. Globally, cancer metastasis is a major cause of morbidity and mortality. In this study, we targeted CXCL12 rather than the chemokine receptor (CXCR4) because most of the drugs failed in clinical trials due to unmanageable toxicities. Until now, no FDA approved medication has been available against CXCL12. Therefore, we aimed to find new inhibitors for CXCL12 through virtual screening followed by molecular dynamics simulation. For virtual screening, active compounds against CXCL12 were taken as potent inhibitors and utilized in the generation of a pharmacophore model, followed by validation against different datasets. Ligand based virtual screening was performed on the ChEMBL and *in-house* databases, which resulted in successive elimination through the steps of pharmacophore-based and score-based screenings, and finally, sixteen compounds of various interactions with significant crucial amino acid residues were selected as virtual hits. Furthermore, the binding mode of these compounds were refined through molecular dynamic simulations. Moreover, the stability of protein complexes, Root Mean Square Deviation (RMSD), Root Mean Square Fluctuation (RMSF), and radius of gyration were analyzed, which led to the identification of three potent inhibitors of CXCL12 that may be pursued in the drug discovery process against cancer metastasis.

## 1. Introduction

The chemokine family consists of relatively small proteins (around 10 kDa) with high potency as chemo attractants [1]. Twenty chemokine receptors have been identified for 50 currently known chemokines. Chemokines are mainly hemostatic or inflammatory and are divided into four different families (C, CC, CXC, and CX3C) on the basis of conserved cysteine residues in the N-terminus [2]. CXCL12 is a CXC hemostatic chemokine and is mainly known as stromal derived factor-1 (SDF-1) [3]. SDF-1 is expressed in several organs and tissues including the liver, testis, lungs, placenta, skin, kidney, pancreas, brain, and colon [4]. CXCL12 is known to have several isoforms, among them, CXCL12-alpha (α), CXCL12-β (beta), CXCL12-γ (gamma), CXCL12-δ (delta), CXCL12-ε (epsilon), and CXCL12-φ (phi) are particularly important. The most expressed isoform in humans is CXCL12-α [5]. CXCL12 expression is enhanced in response to damage caused by toxicities including liver damage, total body irradiation, excessive bleeding, chemotherapy, and tissue injury. CXCL12 has an important physiological role in diverse processes including embryonic development, inflammation, organogenesis, immune mediation, and cancer metastasis [6]. CXCL12-α binds to a specific receptor (CXCR4), a seven transmembrane G-protein coupled receptor that belongs to the G-protein coupled receptor (GPCR) family. CXCR4 is the most common chemokine receptor that is considered to be overexpressed in more than 23 different human cancers [7]. The binding influences intra-cellular signaling pathways including Janus kinase (JNK), mitogen activated protein kinase (MAPK), extracellular signal-regulated kinase (ERK), and protein kinase B (AKT). It plays an important role, in addition to chemotaxis, in cell survival, cellular proliferation, and metastatic spread of cancer cells that express CXCL12, leading to the formation of secondary colonies [8,9,10,11,12,13,14], and a particular role in HIV infection has also been discovered. Cancer cell metastasis contributes to most of the mortality rates. Prevention of cancer cell migration is one of the most paramount medical concerns [15]. The development of novel inhibitors to block the CXCL12–CXCR4 signaling axis could serve as a potential cancer therapeutics. Recently, it has been reported that chemokine neutralization is a successful approach to cancer therapy. Several Tetrazole and Chalcone derivatives have been reported as CXCL12 inhibitors that prevent it from CXCR4 binding, up to some extent [1,16].

Virtual screening (VS) is a standard technique for the identification of novel lead compounds/chemical probes in drug discovery for targeting a receptor of interest. In VS, large libraries of compounds are screened for potential active chemical probes, reducing the size of a large library prior to using other expensive tools [17]. In ligand based virtual screening, potential chemical probes are compared with experimentally identified hits as this technique is based on “the similarity principle”, stating that similar biological responses are produced by similar compounds. Novel potentially active chemical probes are identified using large ligand libraries and by efficiently searching for active compounds nearly similar in chemical properties to the known actives. By using these techniques, potentially active compounds can be proposed, thus reducing the time and expenses of experimental screening of large libraries of compounds in drug discovery [18]. The aim of this study was to identify novel and potential inhibitors of CXCL12 that could be beneficial in limiting the cancer metastasis through various in silico techniques such as virtual screening, molecular docking, and molecular dynamic simulation.

## 2. Results and Discussion

### 2.1. Validation of Docking Software via Re-Docking Run Approach

MOE 2019.0101 was used to validate the co-crystallized ligand pose by the re-docking experiment to check the experimentally determined pose conformation and docking pose. The re-docking result showed minimal RMSD of 0.22 Å.

### 2.2. Pharmacophore Model Generation and Validation

**Figure 1:**
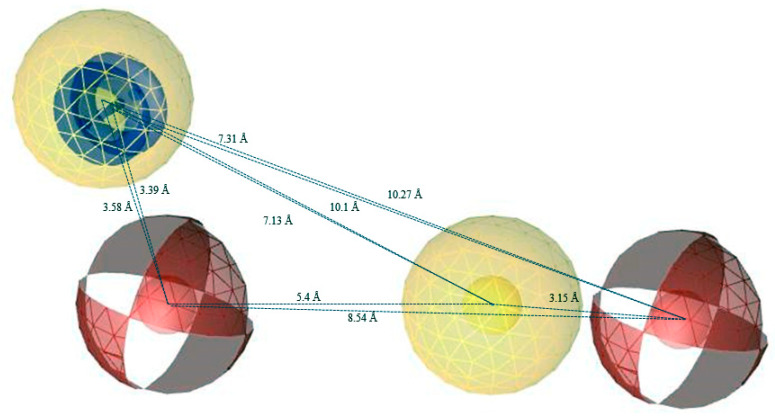
Pharmacophore model geometry and its five features. Yellow represents hydrophobic, blue refers to the aromatic ring, and red denotes the hydrogen bond acceptor feature.

**Figure 2:**
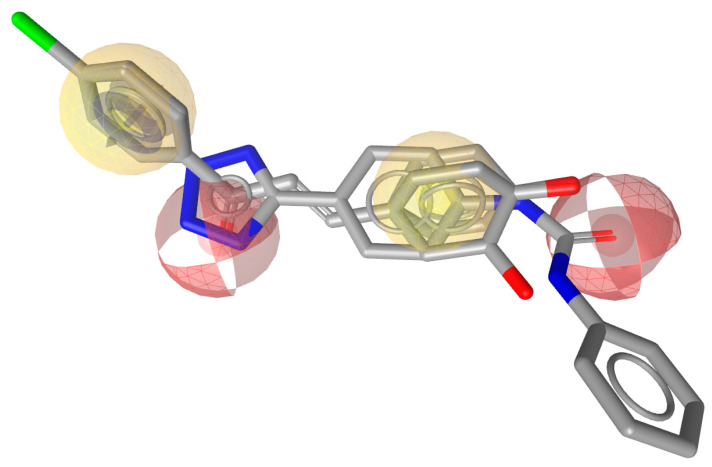
Best alignment of the active compounds previously reported.

One of the most significant applications of pharmacophore model generation is to point out the potent interactions that are involved in binding and increasing selectivity. Ligandscout4.3 software was used for the generation of the pharmacophore model. The ligand-based pharmacophore model was generated on the basis of common chemical features in the active dataset. The pharmacophore model was generated by aligning shared features of different compounds from the active dataset as demonstrated in Figure 1. Random, decoys, actives, and inactive databases were screened against the pharmacophore model generated from shared features of the training dataset (active dataset). Directory of useful decoys (DUDE) [19] is a web0-based program utilized for decoy generation. A maximum five features were found in the final 3D pharmacophore model in the form of two hydrophobic (Hyph), two hydrogen bond acceptors, and one aromatic ring (Aro) feature, as illustrated in Figure 2. The optimum 3D pharmacophore was validated by greater hit-rate in terms of screening active compounds. While screening the random, inactive, and decoy compounds, we observed the lowest hit rate. Table 1 shows all the hit rates of the screening library.

### 2.3. Pharmacophore Based Database Screening

Two libraries ChEMBL and *in-house* were selected for pharmacophore based virtual screening which contain ~1.75 million compounds. The 2D structure of these compounds were converted to 3D and their energy minimization using MMFF94 force field by using Openbabel. Lipinski’s rule of five was applied on the prepared data bases which reduced the databases to 30,669 compounds which were then screened by validated pharmacophore to identify new potent compounds. 1459 hits were retrieved by screening the two data bases from validated pharmacophore. The hits were evaluated further by using Molecular Docking.

### 2.4. Molecular Docking

94 compounds which were retrieved from pharmacophore-based virtual screening were subjected to molecular docking studies to analyze the binding mechanisms. All the compounds were docked into the binding pocket (active site) of the CXCL12 (4UAI). The top ranked conformations of each compound by means of highest docking score were selected. The docking results were further analyzed through protein ligand interaction fingerprint (PLIF) protocol implemented in MOE. PLIF analysis led to finger printing the hot spot active site residues; GLU15, ALA19, ASN22, ASN44, and ARG47 with regards to the ligand interactions. Fifteen out of 94 compounds were selected as hit compounds, which show strong/good binding interaction with the target protein. These top ranked compounds consist of five different classes such as amide, urea, pyridine, piperidine and pyrimidine. Four compounds were selected from amide, urea, pyridine, and 2 from piperidine and pyrimidine for MD Simulation studies.

**Figure 3:**
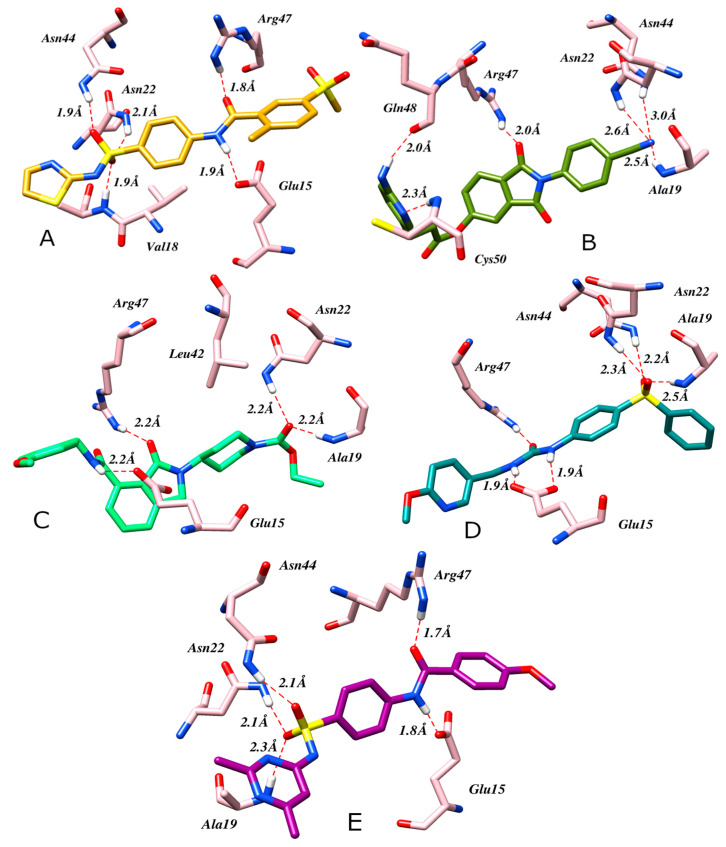
3D model showing interaction of compound CHEMBL1881008 (**A**), CHEMBL1173124 (**B**), CHEMBL1438901 (**C**), CHEMBL2393181 (**D**), and CHEMBL1461227 (**E**).

It was observed from docking conformations that almost all the compounds show strong hydrogen bonding with crucial residues such as GLU15, ALA19, ASN44, and ARG47, while VAL18, and LEU42 form hydrophobic interactions. GLU15 form strong H-bonding with all compounds beside compound 4. ASN44 exhibit strong hydrogen bonding with all the compounds beside compound 16 while ALA19, ASN22, and ARG47 were observed for making strong H-bonding with all the compounds (Supplementary data, Table S1). Besides these some other residues also exhibit interaction with the top hits compounds as shown below in (Table 2) and 3D format (Figure 3). The hits were further subjected to MD Simulation to observe their stability.

### 2.5. Molecular Dynamic Simulation

To further check the stability of the hits along with reference with the cognate ligand of the protein, they were subjected for Molecular Dynamic Simulation (MD) studies. Out of fifteen hits from docking study, one hit from each class were selected for Molecular Dynamic Simulation. 50 ns Molecular Dynamic Simulation runs were performed to elucidate the binding stability, conformational variation and their behavior with the target protein.

### 2.6. Analysis and Visual Inspection of Molecular Dynamic Simulation

The binding mode analysis of all the simulated compounds demonstrated that all the compounds were residing well into the binding pocket of target protein during the course of simulation. Most of the docking interactions were persistent during the 50 ns of simulation. All the simulated compounds observed to mediate hydrogen bonding with GLU15 throughout the whole simulation. Similarly Compound **A**, **B** and **D** establish hydrogen bonding with ALA19, ASN22, ASN44 and ARG47 while Compound C and E observed to establish hydrogen bonding with ALA19 and ARG47. In addition to hydrogen bonding, hydrophobic interactions with VAL18 and LEU42 were also persist throughout the simulation. The time dependent binding interactions demonstrated the potencies of these compounds against CXCL12.

#### 2.6.1. Root Mean Square Deviation (RMSD)

**Figure 4:**
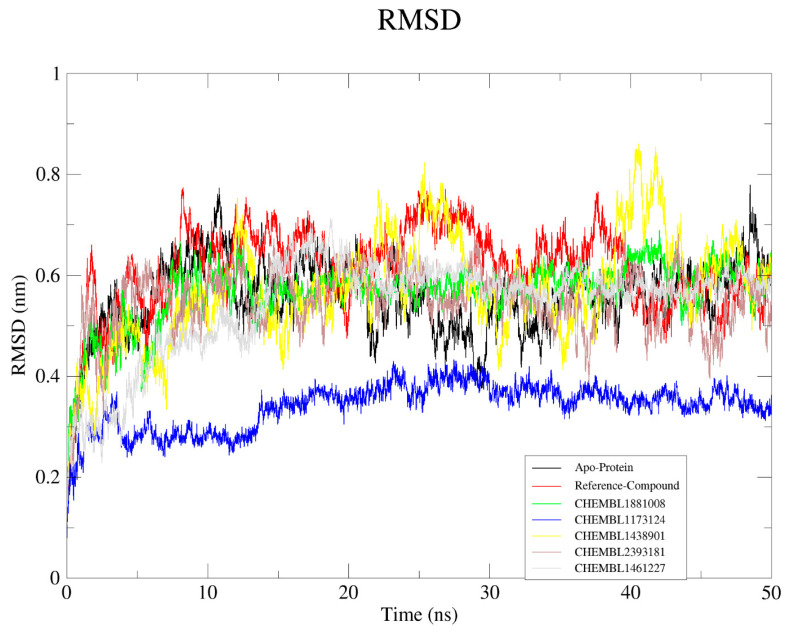
The RMSD graph of all complexes along with apo-protein during 50 ns MD Simulation.

Root mean square deviation is used to determine the backbone differences between the initial structure conformations with its final position. The stability is determined in the form of deviation produced in Molecular Dynamic Simulation. The smaller deviation indicates the more stable complex structure. RMSD of all the system is calculated to evaluate the cα backbone stability for 50 ns simulation. During 50 ns MD run, the average RMSD of Apo protein, reference compound, CHEMBL1881008 (A), CHEMBL1173124 (B), CHEMBL1438901 (C), CHEMBL2393181 (D), and CHEMBL1461227 (E), were found to be 0.55, 0.61, 0.56, 0.33, 0.57, 0.54 and 0.54 Å respectively (Figure 4). The RMSD of compound **A** shows deviation from 0.1 to 0.65 nm in the range of 12 ns after this it is reduced to 0.58 nm till 40 ns and then again its RMSD deviates from 40 to 50 ns due to the slight unfolding of the loops but overall it is quite stable than the Reference compound complex. At the beginning, the RMSD of compound B increase from 0.1 to 0.37 nm in the range of 4 ns, but after that it decreased to 0.28 ns and was consistent till 14 ns, then again the RMSD increased from 0.27 to 0.4 nm and then stabilized throughout 50 ns. While compound **C** showed deviation from 0.1 to 0.88 nm throughout the MD Simulation but its average RMSD is less than the Reference compound complex. Compound **D** RMSD initially increase from 0.1 to 0.72 nm in the range of 18 ns, but afterwards it reduced to 0.6 nm and become constant to 50 ns while the RMSD of compound **E** increased from 0.1 to 0.7 nm throughout the range of 50 ns, like the Reference compound complex during the MD Simulation. The average RMSD of all the hits compound is lesser than the Reference compound complex during 50 ns MD Simulation. Furthermore, it was observed that the hits compound complexes were more stable than the Reference compound complex.

#### 2.6.2. Root Mean Square Fluctuation (RMSF)

**Figure 5:**
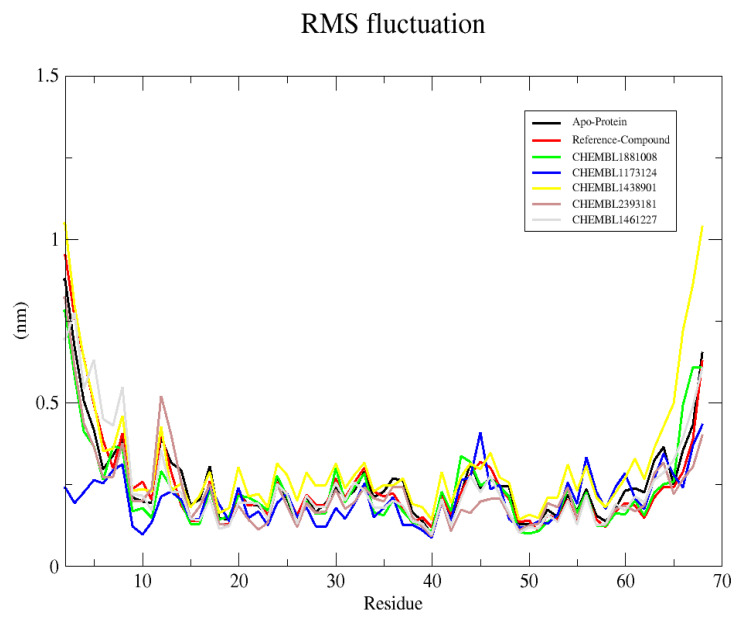
The RMSF graph of all complexes along with apo-protein during 50 ns MD Simulation.

RMSF provides information about the fluctuations per residue of protein-ligand complexes. In protein complexes region of turns, coils and loops show higher fluctuation as compared to beta sheet and alpha helix. The average RMSF values for Apo protein, Reference compound and compounds A-E were found to be 0.26, 0.26, 0.24, 0.20, 0.33, 0.25 and 0.23 nm, respectively as shown in Figure 5. The RMSF values reveal the relative stability of the protein upon binding of virtual hits/compounds. The average atomic fluctuation of the active site residues showed similar binding patterns and reveals that all the compounds stabilize the protein in their adopted conformation for inhibition.

#### 2.6.3. Radius of Gyration

**Figure 6:**
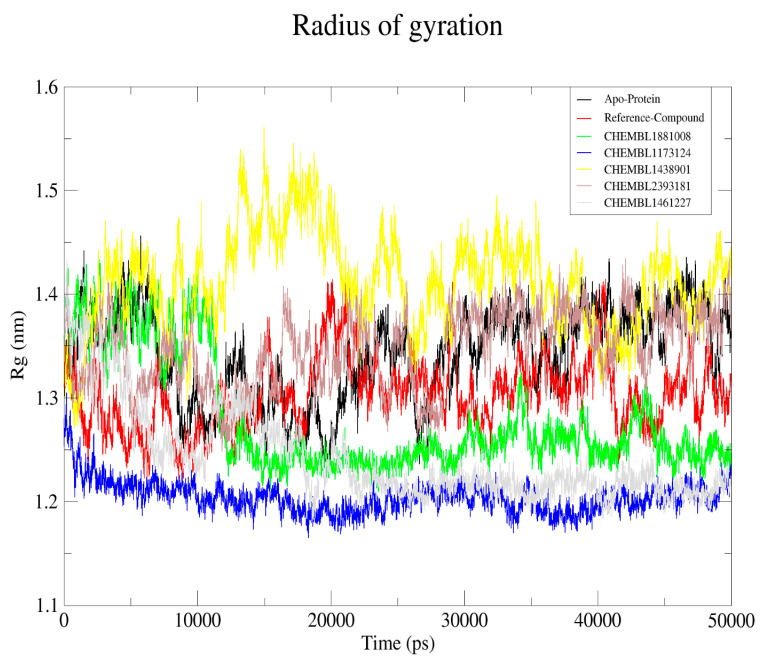
The Radius of Gyration Graph of all complexes along with apo-protein during 50 ns MD Simulation.

The radius of gyration (Rg) was determined to observe the compactness of the protein in the absence and presence of virtual hits. The radius of gyration is defined as the radial distance of mass weighted of atoms from their center of mass. Hence this analysis gives the overall folding and unfolding of protein structure upon binding of virtual hits. The average Rg values calculated for Apo protein, Reference compound, and compounds **A**–**E** were 1.34, 1.30, 1.27, 1.20, 1.41, 1.26 and 1.34 nm, respectively as defined in the Figure 6. The analysis of Rg plot demonstrated that the protein remains compact upon binding of Compound **A**, **B** and **D** during the course of 50 ns of simulation. In contrast, variable uncompactness was observed in the Rg plot of compound **E** and reference compound while Rg value of compound **C** fluctuate more than Apo protein. The results indicate that binding of compound **A**, **B** and **D** significantly stabilized the protein structure.

## 3. Materials and Methods

### 3.1. Preparation of Dataset

**Scheme 1:**
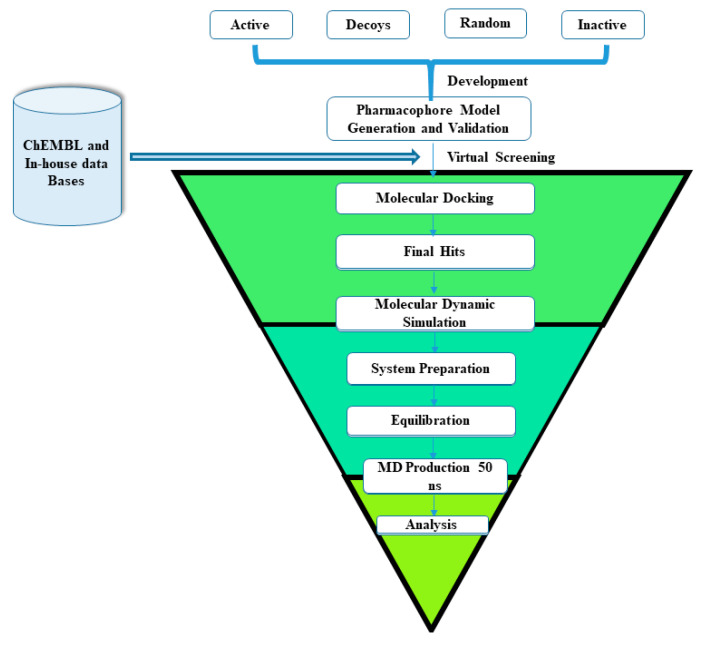
Schematic representation of ligand-based virtual screening and Molecular Dynamic simulation.

**Figure 7:**
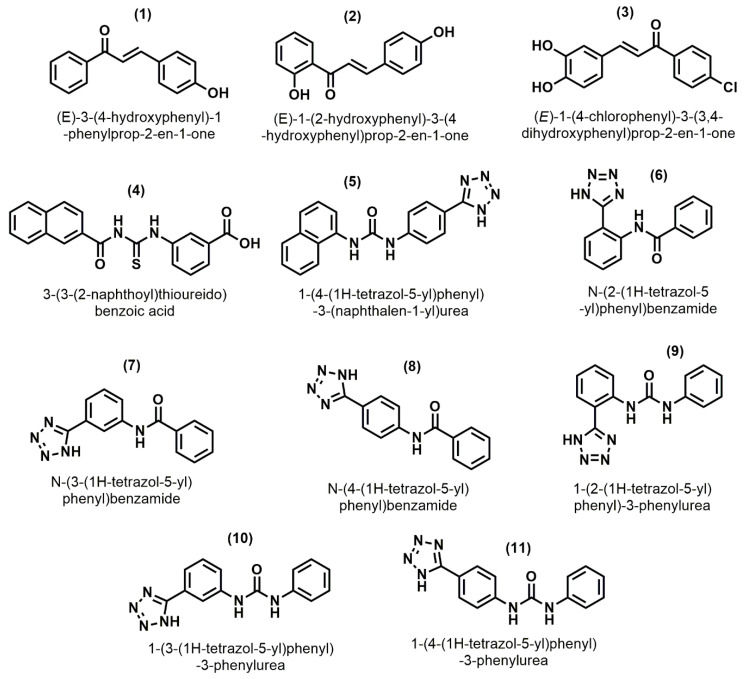
2D Structure of reported inhibitors against CXCL12.

The structure of eleven reported compounds of diverse classes with the reported biological activity against CXCL12 were retrieved from literature as indicated in Figure 7. By using MOE 2019.01, [20] 3D structures of all inhibitors were sketched and prepared using MMFF94 force field for energy minimization [21]. The same protocol for energy minimization and preparation were also used for ChEMBL [22] and *in-house* databases. Over all workflow of virtual screening is depicted in Scheme 1.

### 3.2. Receptor Preparation

X-ray Crystal structure of CXCL12 protein with PDB ID 4UAI [23] was retrieved from protein data bank. It is a homodimer protein comprised of two chains: A and B. ligand was present in chain A, so chain B along with SO4, and water molecules were removed [24]. The 3D structure of target protein was protonated and energy minimized by using AMBER99 force field implemented in molecular operating environment software (MOE).

### 3.3. Re-Docking Experiment

The cognate ligand in the crystal structure extracted and docked back in the binding pocket of protein. Deviation from crystal pose of ligand was analyzed in term of Root mean square deviation to select the docking protocols.

### 3.4. Pharmacophore Model Generation

Ligandscout4.3 Essential [25] were used to generate a 3D pharmacophore model [26]. The most important step in pharmacophore model generation is to select suitable chemical features e.g., HBA (hydrogen bond acceptor), HBD (hydrogen bond donor), Aro (aromatic ring) and Hyph (hydrophobic) in training set. Chemical features present in training set molecules were consider for mapping pharmacophore model generation. All the shared feature of training set molecules was aligned and assembled together for generation of final pharmacophore model. In final pharmacophore model 5 features were present. Pharmacophore model performance and quality was validated from its ability of distinguish between decoys, inactive random and active compounds.

### 3.5. Pharmacophore Validation

Validation of pharmacophore model were done by screening entire ligand data base file such as decoys, random, actives and inactive [27]. Using pharmacophore fitness score function in LigandScout4.3, were utilized to ranked the poses of compounds. For Validation of pharmacophore model, its ability to discriminate between decoys, random, actives and inactive data sets was evaluated. All the four data sets of compounds were screened against the selected pharmacophore model which gave a genuine hit-rates.

### 3.6. Pharmacophore-Based Virtual Screening

Two libraries such as the ChEMBL and *in-house* databases were screened against the validated pharmacophore model. The two main purposes of virtual screening was (1) the validation of the pharmacophore model with the help of known inhibitory activity of compounds, and (2) finding new drug like compounds that may be potent for further assessment. Lipinski’s rule of five [28] was applied to retrieved drug like hit compounds. Ninety-four hit compounds were retrieved by screening from the validated pharmacophore model.

### 3.7. Molecular Docking

Pharmacophore screened compounds were further subjected for docking into the active site of CXCL12. Docking studies were performed by using the default parameter implemented in the Molecular Operating Environment (MOE) program 2019.01 [29]. Form docking hit compounds and top best pose on the basis of docking score were selected for further evaluation. The binding pose of protein and hit compounds were visualized by using MOE.

### 3.8. Molecular Dynamic Simulation

To further observe the stability of the hit compounds in the binding pocket, molecular dynamic simulation of the respective and reference compound was done by using GROMACS-2018 [30] by employing the GROMACOS9653a6 force field [31,32]. The topologies of the ligand were generated through Antechamber Python Parser interface (ACPYPE) [31,33]. Counter ions were added to neutralize the SPCE water model present in the cubic box. The initial structure was clean from unwanted contacts by performing the energy minimization using steepest descent algorithm, which was followed by Constant Number of particles, Volume and Temperature (NVT) and Constant Number of particles, Pressure and Temperature (NPT) equilibration steps [34]. The counter ions along with the solvent molecules were allowed to simulate restraining the back bone of the protein. Both steps were executed by 20 ns at a pressure of 1 bar and 300 K, respectively. The Berendsen thermostat and barostat were employed to maintain the pressure of the system [35,36]. The atoms involved in hydrogen bonding and geometry of the water molecule were constrained employing Linear Constraint Solver (LINCS) and SETTLE, respectively [37]. Long-range electrostatic interaction was calculated through the particle mesh Ewald (PME) method [37,38]. The equilibrated structures were subjected to production Molecular Dynamic (MD) for 50 ns and the results were visualized by VMD [36].

## 4. Conclusions

CXCL12 is an important target in cancer metastasis and up until now, there has been no FDA approved drug available in the market for its inhibition. In this study, pharmacophore based virtual screening and molecular docking, along with molecular dynamic simulations, were performed to discover new hits and investigate the binding conformation and stability of the target protein. The generated pharmacophore model had two hydrogen bond acceptors, two hydrophobic, and one aromatic feature. Different test sets were used to validate the pharmacophore model, and subsequently screen two databases from it. A total of 94 compounds were retrieved from the pharmacophore-based virtual screening and docking. A further fifteen compounds were selected on the basis of docking score and significant protein ligand binding interaction. Finally, five compounds were selected for molecular dynamic simulations in which the RMSD, RMSF, and Rg results revealed that these compounds significantly stabilize the protein upon binding. In conclusion, compounds A, B, and D can act as promising leads in the development of potent inhibitors against CXCL12 to reduce cancer metastasis.

## Figures and Tables

**Table 1:** The hit rate of all four libraries in the pharmacophore model validation.

S. No	Libraries	Total No	Picked Out	Hit Rate%
1	Actives	11	8	72.78
2	Inactive	05	0	0
3	Decoys	550	20	3.61
4	Random	115	18	15.65

**Table 2:** Molecular interactions between protein-ligand complexes.

Compound ID	Docking Score	H-Bonds	Hydrophobic	Salt Bridges	Pi-Stacking
**Reference compound**	−5.970	Glu15, Ala19, Asn22, Asn44, Arg47	Val18, Leu42	----	----
**CHEMBL1881008** **(A)**	−6.1680	Glu15, Ala19, Asn22, Asn44, Asn45, Arg47	Val18, Leu42, Val49	----	----
**CHEMBL1173124** **(B)**	−6.1645	Ala19, Asn22, Asn44, Arg47, Gln48, Cys50	Phe13, His17	----	----
**CHEMBL1438901** **(C)**	−5.8470	Glu15,Ala19, Asn22, Arg47	Leu42	----	----
**CHEMBL2393181** **(D)**	−5.6232	Glu15, Ala19, Asn22, Asn44, Arg47	----	----	----
**CHEMBL1461227** **(E)**	−5.2401	Glu15,Ala19, Asn22, Asn44, Arg47	Ala19	----	----

## Data Availability

The datasets can be obtained with justification from the corresponding author.

## Supplementary Materials

The following are available online. Table S1: Molecular interactions between protein-ligand complexes of all screened compounds.

## Abbreviations

CXCL12	C-X-C motif chemokine 12
CXCR4	C-X-C chemokine receptor type 4
SDF-1	Stromal Derived Factor-1
JNK	Janus kinase
MAPK	Mitogen Activated Protein Kinase
RMSD	Root Mean Square Deviation
RMSF	Root Mean Square Fluctuation
Rg	Radius of gyration
VS	Virtual Screening
ERK	Extracellular Signal-Regulated Kinase
AKT	Protein kinase B
MMFF94	Merck Molecular Force Field 94
PLIF	Protein Ligand Profiling Fingerprinting
NVT	Constant Number of particles, Volume and Temperature
NPT	Number of Particles, Pressure and Temperature
LINCS	Linear Constrain Solver
PME	Particle Mesh Ewald
