# Supplementary material for: Discovery of Potential Chemical Probe as Inhibitors of CXCL12 Using Ligand-Based Virtual Screening and Molecular Dynamic Simulation
Source: Molecules. 2020 Oct 20;25(20):4829. doi: 10.3390/molecules25204829 (PMC7594044; doi:10.3390/molecules25204829)
Supplement: Supplementary file 1 [file molecules-25-04829-s001.zip › molecules-967518-supplementary.docx]

**Supplementary Materials**

**Discovery of potential chemical probe as inhibitors of CXCL12 using Ligand-Based Virtual Screening and Molecular Dynamic Simulation**

**Sajjad Haider^1^,** **Assem Barakat*^2,3^, and Zaheer Ul-Haq*^,1^.**

^1^ Dr. Panjwani Center for Molecular Medicine and Drug Research, International Center for Chemical Sciences, University of Karachi, Karachi-75270, Pakistan. [sajjadrealist@gmail.com](mailto:sajjadrealist@gmail.com) (S.H.).

^2^ Department of Chemistry, College of Science, King Saud University, P. O. Box 2455, Riyadh 11451, Saudi Arabia.

^3^ Department of Chemistry, Faculty of Science, Alexandria University, P.O. Box 426, Ibrahimia, Alexandria 21321, Egypt.

***** Correspondence: [zaheer_qasmi@hotmail.com](mailto:zaheer_qasmi@hotmail.com); [zaheer.qasmi@iccs.edu](mailto:zaheer.qasmi@iccs.edu) (Z.U-H).; [ambarakat@ksu.edu.sa](mailto:ambarakat@ksu.edu.sa) (A.B.) ; Tel.: +966-11467-5901(A.B.); Fax: +966-11467-5992(A.B.).

**Table S1:** Molecular interactions between protein-ligand complexes of all screened compounds.

| **Compound ID** | **Docking Score** | **H-Bonds** | **Hydrophobic** | **Salt Bridges** | **Pi-Stacking** |
| --- | --- | --- | --- | --- | --- |
| **Reference compound** | -5.970 | Glu15, Ala19, Asn22, Asn44, Arg47 | Val18, Leu42 | ---- | ---- |
| **CHEMBL3237006** | -6.3090 | Arg12, Phe13, Glu15, Ala19, Asn22, Asn44, Arg47 | ----- | ---- | ---- |
| **CHEMBL1468113** | -6.2012 | Glu15, His17, Ala19, Asn22, Asn44, Arg47 | ----- | Arg47 | ---- |
| **CHEMBL1881008** | -6.1680 | Glu15, Ala19, Asn22, Asn44, Asn45, Arg47 | Val18, Leu42, Val49 | ---- | ---- |
| **CHEMBL1173124** | -6.1645 | Ala19, Asn22, Asn44, Arg47, Gln48, Cys50 | Phe13, His17 | ---- | ---- |
| **CHEMBL1441103** | -6.1336 | Glu15, His17, Ala19, Asn22, Asn44, Arg47, | Phe13, His17 | ---- | ---- |
| **CHEMBL1438901** | -5.8470 | Glu15,Ala19, Asn22, Arg47 | Leu42 | ---- | ---- |
| **CHEMBL2393181** | -5.6232 | Glu15, Ala19, Asn22, Asn44, Arg47 | ---- | ---- | ---- |
| **CHEMBL1375785** | -5.4192 | Glu15, Ala19, Asn22, Asn44, Arg47 | Arg47 | ---- | ---- |
| **CHEMBL1311599** | -5.3073 | Glu15, Ala19, Asn22, Asn44, Arg47 | ---- | ---- | ---- |
| **CHEMBL3660721** | -5.2618 | Ala19, Asn22, Asn44, Arg47 | ---- | ---- | Phe13 |
| **CHEMBL1461227** | -5.2401 | Glu15,Ala19, Asn22, Asn44, Arg47 | Ala19 | ---- | ---- |
| **CHEMBL3582349** | -5.1736 | Glu15, His17, Ala19, Asn22, Asn44, Arg47 | Val18 | ---- | ---- |
| **CHEMBL3582348** | -5.1508 | Glu15,His17,Ala19, Asn22, Asn44, Arg47 | Val18 | ---- | ---- |
| **CHEMBL479089** | -5.1488 | Glu15, His17, Ala19, Asn22, Asn44, Arg47 | Val18 | ---- | ---- |
| **CHEMBL1338251** | -4.8792 | Glu15, Ala19, Asn22, Asn44, Arg47 | ---- | ---- | ---- |
